# Specific Hygiene Procedures and Practices Assessment: A Cross-Sectional Study in Fresh Fishery Product Retailers of Lisbon’s Traditional Food Markets

**DOI:** 10.3390/foods10081805

**Published:** 2021-08-04

**Authors:** Rafael Sousa Oliveira, Maria José Rodrigues, Ana Rita Henriques

**Affiliations:** 1 CIISA—Centro de Investigação Interdisciplinar em Sanidade Animal, Faculdade de Medicina Veterinária, Universidade de Lisboa, 1300-477 Lisboa, Portugal; rafael.sousa.oliveira@hotmail.com; 2 Departamento de Estruturas de Proximidade e Espaço Público, Direção Municipal de Economia e Inovação da Câmara Municipal de Lisboa, 1600-036 Lisboa, Portugal; maria.jose.rodrigues@cm-lisboa.pt

**Keywords:** fishery products retail, food handlers, food safety, hygiene conditions, hygiene practices, training

## Abstract

This study aimed to assess the compliance of specific hand hygiene and food contact surfaces hygiene in fresh fishery product retailers (FFPR) and its relation to socio-demographic characteristics that might influence the observed procedures and practices. A cross-sectional study was conducted in traditional food markets’ FFPRs (*N* = 74) using interviews and audits for data collection purposes. Results revealed that women were overrepresented among FFPR managers; most were over 50 years of age and had a long working experience in fish retail activities, despite a low educational level. The majority had attended training courses in food hygiene, safety, and Hazard Analysis and Critical Control Points-based procedures. Both documental assessment and hand hygiene procedures and practices verification revealed a low level of compliance. Many documents supporting hygiene practices were lacking. Several non-conforming requisites were identified related to the handwashing procedure and equipment and to glove wearing practices. A higher level of compliance was obtained in food contact surfaces hygiene procedures and practices verification, with complete hygiene procedures applied and proper cleaning of utensils and chemical products management in several cases. These findings reflect an ambiguous understanding and misconceptions related to hygiene procedures and practices, suggesting the need to improve and update FFPR food handler training regarding basic food hygiene and safety.

## 1. Introduction

According to the World Health Organization (WHO), over 200 diseases are caused by the consumption of food contaminated with bacteria, viruses, parasites, or chemical substances [1]. Fish and fishery products were the food vehicle of 27% of the total strong-evidence foodborne outbreaks reported by European member states in 2019, representing a total of 193 outbreaks involving 1610 cases of disease. Considering causal agents, norovirus and other calicivirus were the leading agents, followed by histamine and marine biotoxins [2]. Unprocessed contaminated ingredients and contamination by food handlers have been pointed out as leading causes for foodborne disease outbreaks in European member states [2]. More specifically, cross-contamination events and insufficient or inadequate food processing temperatures were outlined as major contributing factors [2]. Food handlers play a critical role in food mishandling, and in the retail sector, cross-contamination prevention is of upmost importance.

Currently, European food establishments must comply with the strict general food hygiene and safety requirements detailed in Regulation (EC) No 852/2004 [3], supplemented by specific hygiene requirements on food of animal origin, provided in Regulation (EC) No 853/2004 [4]. In local retail establishments directly supplying the final consumer, prerequisite programs, together with Hazard Analysis and Critical Control Points (HACCP) based procedures, can be appropriate and sufficient to adequately control hazards [5], as is the case with fresh fishery product retailers (FFPR) in Lisbon’s traditional food markets. 

Prerequisite programs are a structured approach based on good hygiene practices, including, among other things, premises cleaning and sanitizing and personal hygiene. Food processing equipment and surfaces should be able to be cleaned and sanitized to an acceptable level. The selection of appropriate cleaning equipment and chemicals, together with an appropriate cleaning and sanitizing program, are essential in food establishments [6]. The combination of concentration, mechanical action, time, and temperature is of major importance for efficient cleaning and sanitizing [7]. Additionally, these practices need to be closely monitored, verified, and validated to obtain better results over time [8,9]. Simultaneously, food handlers are required to maintain high standards of personal hygiene regarding protective clothing and equipment, handwashing procedures, and general behavior towards food hygiene and safety [10,11]. Hand hygiene is an essential part of a personal hygiene program in food establishments. Handwashing compliance has been associated with a reduction of cross-contamination events, especially if consistently and regularly applied [12]. The recommended handwashing procedure urges a minimum of 40 to 60 s of handwashing duration, with about 30 s of hand friction with soap. A hand sanitizer may be used as a final step to reduce microbial populations [13]. Additionally, wearing gloves, whether disposable or reusable, can be an effective way to prevent cross-contamination of food from hands; still, improper glove wearing can increase this risk [14].This is especially relevant considering FFPR processing operations, as various tasks, such as fish gutting and spine and scale removal, promote glove deterioration. In this way, prerequisite programs provide a foundation for HACCP-based procedures implementation by enabling control of operational conditions within a food establishment and promoting favorable environmental conditions for the hygienic and safe production of food. HACCP-based procedures are risk-based and systematic, identifying and assessing specific hazards while establishing control systems focusing on prevention [5].

In relation to food hygiene and safety, food handlers must receive appropriate training and be fully informed and conscious of their responsibilities. According to Regulation (EC) No. 852/2004 [3], food business managers must also receive training in HACCP. Training updates are important to assure that the proper information is being transmitted, improving hygiene practices among food handlers [5,10,15]. As such, a manager’s commitment and staff engagement are crucial for the successful implementation of prerequisites programs and HACCP-based procedures [16]. The retail sector, and FFPR in particular, have been poorly characterized on the implementation of good hygiene practices. This study aimed to assess the compliance of FFPR with specific hand hygiene and food contact surfaces hygiene practices and its relation with socio-demographic characteristics that might influence the observed procedures and practices.

## 2. Materials and Methods

### 2.1. Study Design and Participants

A cross-sectional study was conducted from October 2020 to April 2021 to assess hygiene procedures and practices among food handlers in fresh fishery product retailers (FFPR) of Lisbon’s traditional food markets. FFPR were defined as establishments that sell or supply fresh and prepared fishery products to the final consumer, according to Regulation (EC) No. 853/2004 [4] definitions. All of the FFPR (*N* = 74) in business in Lisbon’s traditional food markets were previously informed of the study aim and methods, and were asked to participate voluntarily during regular working days.

### 2.2. Fresh Fishery Product Retailers’ Assessment

For data collection purposes, each FFPR assessment included: (i) a face-to-face interview to collect business manager’s socio-demographic data; (ii) an audit to assess hand hygiene and food contact surfaces hygiene procedures and practices.

#### 2.2.1. Interview for Socio-Demographic Data Collection

A questionnaire was designed and applied to each FFPR business manager for socio-demographic data collection purposes. To characterize food handlers, interviews included questions on age, sex, education level, participation in food hygiene and safety training, and years of work experience as a food handler in fresh fishery products retail establishments.

#### 2.2.2. Auditing Hand Hygiene and Food Contact Surfaces Hygiene Procedures and Practices

A checklist was specially prepared to audit good hygiene practices, together with the implemented food safety management system (FSMS) in each of the participating FFPR. The checklist included 41 requisites (close-ended questions), grouped in three sections to evaluate procedures and practices concerning hand hygiene and food contact surfaces hygiene (Appendix A). The requisites were based on Codex Alimentarius food hygiene basic texts [17], Regulation (EC) No. 852/2004 on the hygiene of foodstuffs [3], and the Good Hygiene Practices Guide for Fresh Fishery Products [18]. The checklist was pre-tested in four FFPR, and the necessary adjustments were done to enhance data collection. The audit was performed during a regular working day, in which food handlers were observed throughout the process of food handling and retail sale activities. Because FFPR managers were also food handlers in all of the considered establishments, managers were sampled to represent food handlers in some particular requisites linked to personal hygiene.

### 2.3. Data Analysis

Questionnaire and audit checklist data were tabulated and described using MS Excel 2016 software (Microsoft Corporation, Redmond, WA, USA). For data analysis purposes, R software version 3.5.1 (R Development Core Team, Vienna, Austria) was used, and Fisher’s exact test was selected to check for significant (*p* < 0.05) association between categorical variables.

## 3. Results and Discussion

### 3.1. Fresh Fishery Product Retailers Socio-Demographic Data

All of the fresh fishery product retailers (FFPR) in business (*N* = 74) in 18 traditional food markets in Lisbon agreed to participate and were included in this study. A total of 147 food handlers were employed in these establishments (Table 1). The participating FFPR had a median of one food handler, although some presented seven food handlers, being classified as microenterprises [19].

All the managers of the participating FFPR (*N* = 74) had Portuguese as their primary language, and the vast majority had Portuguese nationality (97.3%). Women represented the predominant sex (75.7%) among FFPR managers (Table 2). These results are in line with those reported in other studies addressing food handlers in different food service establishments, in which women were prevailing [20,21,22]. Cunha et al. [20] discussed that the predominance of women working as food handlers might be related to social inheritance. Both men and women engage in fish retail activities but, traditionally, while men’s role is related to fish harvesting, women play a great role in post-harvest activities [23].

The majority (67.5%) of the participants was more than 50 years old (Table 2), disclosing a prevailing aged population among FFPR managers of Lisbon’s traditional food markets consistent with other reported results [24,25]. Contrastingly, similar studies conducted in Brazil, China, and Malasia concluded that the majority of food operators in those countries belonged to younger age groups [26,27,28]. FFPR managers were also revealed as being fresh fishery product professionals for several years, with 77.1% of the respondents working for at least 21 years in this activity (Table 2). Similar results were reported for Argentinian food handlers, of which 30% had worked for more than 15 years in fishing establishments [29]. Most of the respondents (77%) also were also confirmed as employing at least one or two food handlers in their FFPR business, while 6.8% had five or more food handler employees. In many cases, relatives were a significant share of the staff, and several FFPR had inherited the business from their parents or close relatives. These results were not surprising, as Portuguese food businesses are still embedded in a familiar character [30,31]. 

As for the educational level, FFPR managers presented limited education, with 74.3% of the respondents having completed the 1st cycle (primary education), while 6.8% were illiterate (Table 2). The predominance of a low education level is clear and has also been identified in other studies assessing food handlers of Lisbon´s traditional food markets and other food services [25,26]. Food handlers are not required by law to have advanced levels of education beyond training in food hygiene and safety [20]. The familiar character of these food businesses aggregates workers that are not selected based on their education level or vocational training experience. Considering training in food hygiene and safety and HACCP-based procedures, 6.8% (*n* = 5) FFPR managers never attended a training course. These results are similar to 2.3% (*n* = 2) reported among food handlers in butcher’s shops in Portugal [11], but lower than 20.4% (*n* = 43) reported for fish handlers in Argentinean fish establishments [29], and 31.7% and 85.5% reported for food handlers from Brazilian food service establishments [20] and in fast food restaurants in Jordan [32], respectively.

Most FFPR managers (63.5%) attended a training course in the previous 5 years (Table 2). However, most managers of FFPR establishments that contracted a food safety consultancy company (28.4%; *n* = 21) had attended annual training courses in food hygiene and safety and HACCP-based procedures. Regular and continuous training in food hygiene and safety is widely recognized as an improvement tool, contributing to an increase in food safety procedures and practices awareness among food handlers [15,27,29,33,34].

### 3.2. Fresh Fishery Product Retailers Audit Assessment

#### 3.2.1. Documental Assessment of Hand Hygiene and Food Contact Surfaces Hygiene

Each audit of FFPR establishments included an assessment focusing on hand hygiene and food contact surfaces hygiene supporting documents. For this purpose, as shown in Appendix A, hygiene plans, work instructions, and technical and safety data sheets of in-use detergents and sanitizers were verified. Available and easily displayed work instructions enhance hygiene practices in food establishments, as food handlers tend to follow them thoroughly [35,36,37,38,39,40]. Hand hygiene specific document compliance frequencies were low (Table 3), due to the lack of hand soap or hand sanitizer specification, or to inexistent work instructions, not addressing best practices recommendations [5,18]. Although two (2.7%) FFPR presented all the requested documents, the hand soap that was being used did not match with the presented technical and safety data sheets (Table 3).

Food contact surfaces hygiene specific document compliance frequencies were also low (Table 3). Most FFPR (91.9%; *n* = 68) were not able to present any food contact surfaces hygiene related documents, and only two FFPR presented all the required documents. In four FFPR, no detergents and sanitizers were mentioned as part of the hygiene procedure for food contact surfaces, and no technical and safety data sheets were presented, failing to comply with food safety and occupational safety requirements [5,18,38]. The communication of chemical substance hazards plays an important role in ensuring a safe use of soaps, detergents, and sanitizers, among other chemical substances used in food-related environments, both in professional and at-home scenarios [41].

In small-scale food business operators, such as FFPR in traditional food markets, prerequisite programs, together with HACCP-based procedures, should be considered for hazard control; these are supported by the commensurate documents and records, including, not exclusively: applied prerequisites, work instructions, standard operational procedures, and control instructions [5]. Carefully selected documents play a key role in food safety management system maintenance and application [42]. Although good hygiene practices and HACCP-based procedures have been internationally accepted and approved [3], and facilitation arises from European Commission Notice 2016/C 278/01 [5], small-sized companies still reveal problems with its practical application and implementation [16,42]. Moreover, written documents are not part of these companies’ culture, being considered unnecessary and dispensable [16]. Still, documents and records are important tools to verify the proper functioning of the food safety management system by official control authorities [5].

#### 3.2.2. Hand Hygiene Procedures and Practices Verification

Most FFPR managers (58.1%; *n* = 43) presented suitable hand and nail hygiene for food handling, with no apparent lesions or wounds, and 28.4% did not display hand or wrist accessories, such as watches, bracelets, or rings, including wedding rings (Table 4), in accordance to mandatory requirements [3] and best practices [5,18].

Regarding an existing handwashing sink, 25.7% (*n* = 19) of the FFPR presented an exclusive handwashing sink and four of these had non-manual water control faucets, observing mandatory requirements [3]. Still, hot water was not available in 73% (*n* = 54) of the audited FFPR, failing to comply with legal requirements [3] and also with hand washing best practices [5,18]. A work instruction illustrating all the necessary steps for a complete handwashing procedure in the vicinity of the handwashing sink was evidenced in 9.5% (*n* = 7) of the studied FFPR (Table 3). All these non-conforming requisites negatively affect the handwashing effectiveness in these establishments [14,17]. Santos et al. [11] assessed Portuguese butcher shops and found that 17.8% did not have hot water in the handwashing sinks, while 9.6% presented manual control faucets, demonstrating that butcher shops display a higher compliance level in these requisites.

A complete and appropriate handwashing procedure involves using lukewarm water, meticulous hand rubbing with soap for approximately 30 to 40 s, hand rinsing under running water, and hand drying with a disposable paper towel [3,17]. Considering the observed handwashing procedure carried out by food handlers during the audit assessment, in 66.2% (*n* = 49) of the cases running water was used to perform the task. Soap use was scarcely observed, and the recommended handwashing soaping and friction time—30 to 40 s—was not achieved (Table 4). In several cases, handwashing consisted of wetting hands in cold water, which constitutes in itself a misconception of the handwashing procedure. In other works investigating food workers handwashing procedures, 72.5% of food handlers from Dubai’s catering establishments were reported to wash their hands properly, using hot water and soap [43], and 60% of the handwashing practices were adequate among Spanish catering food workers [44]. Considering the hand drying step, only 18.9% (*n* = 14) performed it correctly using disposable paper towels (Table 4), observing recommended best practices [5]. Cloths were also used for hand drying purposes, but in most cases these were in poor condition—wet, worn, or used by other food handlers or in other tasks, constituting an important cross-contamination vehicle. These practices reveal a lack of knowledge on food hygiene and safety matters, as cloths are frequently associated with cross-contamination events in food establishments, some of which have been linked to foodborne outbreaks [45,46,47,48]. Hand sanitizer was not used in any of the studied FFPR establishments. Hand sanitizer use is not essential, as in most food handling activities proper handwashing using lukewarm water and liquid soap is enough to reach a proper hand hygiene level [49].

Glove wearing was observed in 47.3% (*n* = 35) of the establishments, with 29 FFPR opting for reusable gloves and six for disposable gloves. Before and/or after putting on gloves, no handwashing was performed, as mentioned in European Commission Notice 2016/C 278/01 [5], and similar results were obtained in other studies [50]. In our study, it was noticed that reusable gloves were placed in inappropriate places, such as pipes or faucets, after use, failing to observe the mandatory requirements in Commission Regulation (EC) No 852/2004 of 29 of April 2004 [3]. Disposable gloves were only changed when visibly deteriorated and/or in poor condition. The main aim of glove wearing in food establishments is to minimize physical, chemical, and microbiological contamination of foodstuffs. However, gloves may offer a false sense of hygiene to food handlers, especially when they are not trained and glove wearing is not properly supervised [51]. The glove wearing attitudes and practices observed in this study are consistent with the ones reported by Green et al. [52] and Soares et al. [26], in which food handlers considered gloves as personal protective equipment instead of a food protection barrier. This study’s observations underline the need for proper, regular, and updated training of FFPR food handlers in good hygiene practices [11,15,26,51,52] to raise awareness of proper hand hygiene in order to reduce the risk of food contamination. Training methodologies should be adapted to the trainee’s level of education, and could include on-the-job training as an effective way to bring theoretical knowledge into practice [29,53].

#### 3.2.3. Food Contact Surfaces Hygiene Procedures and Practices Verification

All of the FFPR premises presented food contact surfaces composed of innocuous and washable materials (Table 5), in accordance with Commission Regulation No 852/2004 food hygiene requisites [3]. Stainless steel and high-density polyethylene were the most common materials in fish display counters, weighing equipment, cutting boards, fish labels, knifes, and other preparation utensils, such as fish scalers, thus enabling effective hygiene procedures, as these materials are resistant to cleaning and sanitizing substances [53]. Nevertheless, some knifes and scalers presented wooden handles, compromising microbial reduction or elimination during hygiene procedure application. In a study focusing on the efficiency of hygiene procedures used in food contact surfaces [54], the authors concluded that hygiene procedures were more effective on stainless steel and high-density polyethylene surfaces, in contrast with wooden materials. With respect to food contact surfaces maintenance, most were also found to be in good condition (Table 5), which can be related to their being composed of materials that are fairly resistant and durable [55,56].

In only 20 FFPR (27%) was a complete hygiene procedure observed being applied to food contact surfaces [49]. For that, surfaces were pre-rinsed with water, then detergent was applied onto the surface and rubbed using a cleaning utensil, then the surface was rinsed again with water and a sanitizer was applied, followed by a final rinsing step [5]. The cleaning and sanitizing steps used in food contact surfaces hygiene procedures, though distinct and independent, have a complementary action [17]. Prior to sanitizing, surfaces should not contain any components that can inactivate the sanitizer, such as organic matter residues, due to its neutralizing effect [7]. This is very important to prevent microbial growth and related cross-contamination events. Some FFPR (*n* = 13) solely used detergent, while others (*n* = 5) chose to use a sanitizer alone. The use of sanitizer alone is least effective when compared to the exclusive use of a detergent on food contact surfaces [54]. Although incorrect from a hygienic standpoint, other authors have also reported the sole use of sanitizers in food contact surfaces of restaurant kitchens [57]. In 47.3% (*n* = 35) of the hygiene procedures, an arbitrary mix of the detergent and sanitizer was prepared to be used in food contact surfaces, without considering the recommended instructions. Mixing detergents and sanitizers reduces the efficiency of the sanitizing step [58].

Regarding the used chemicals in food contact surfaces hygiene procedures, liquid detergent for manual dish washing was the preferred choice (82.8%; *n* = 53), while traditional chlorine bleach was the most applied sanitizer (70.3%; *n* = 45). Among food-grade sanitizers, chlorine compounds are the most affordable and popular sanitizers [59,60,61]. Cleaning utensils were used by 65 FFPR (87.8%), with the remaining operators using solely water pressure and/or the physical action of the food handler’s hands. Among the used utensils, scouring pads were the most used, followed by cloths and plastic scrubbing brushes (Table 5).

The majority of the assessed FFPR (81.1%; *n* = 60) kept chemical products (detergents and sanitizers) in their original packages, with well preserved and readable (*n* = 52) labels, enabling a proper preparation and use of these chemical products, according to food safety and occupational safety requirements [5,18,38]. This is particularly relevant, because, as abovementioned, no technical or safety data sheets were available in most cases (Table 3). These products were stored in an identified closed cupboard (*n* = 22) and kept away from food handling areas, revealing a proper storage of chemical products (Table 5).

No significant statistical association was found between food contact surfaces hygiene procedures and education level, years of employment in FFPR, and training in hygiene and food safety, besides the age and sex of the participants (*p* > 0.05).

## 4. Conclusions

The assessed FFPR were microenterprises employing a median of one food handler, and presenting a familiar character in several cases. Most managers were women, with more than 50 years of age, low educational level, and long working experience in fish retail activities. The majority had attended a training course in food hygiene, safety, and HACCP-based procedures in the previous 5 years.

Both documental assessment and hand hygiene procedures and practices verification revealed a low level of compliance. Most of the requested documents supporting hygiene practices were not presented. Several non-conforming requisites were identified related to handwashing equipment, glove wearing, and with the handwashing procedure itself, that consisted of wetting hands in water, revealing an incorrect practice. A higher level of compliance was obtained in food contact surfaces hygiene procedures and practices verification. In several cases, a complete hygiene procedure was applied, with proper cleaning utensils and chemical products being used; nevertheless, some FFPR used sanitizer alone in food contact surfaces hygiene procedures.

Taken together, the obtained results reflect misconceptions and an ambiguous understanding of the purpose of hygiene procedures and practices. The traditional and family-embedded character of these FFPR, together with the low level of education and training recycling, perpetuates incorrect practices, suggesting the need to improve managers’ and food handlers’ attitudes towards hygiene. Although official control authorities frequently assess the compliance of food safety requirements, food business operators are the ones responsible for the daily management of their food safety management systems. In this way, regular training assumes an essential role in the improvement of food safety and hygiene practices among food handlers. The training program should include basic food hygiene and safety procedures and practices, and considering the assessed population, an on-the-job training format would be most appropriate.

## Figures and Tables

**Table 1:** Food handler distribution in fresh fishery product retailers of Lisbon´s traditional food markets.

Market	No. FFPR	Food Handlers		
		Total	Minimum–Maximum Range	Median
A	6	11	1	1
B	1	1		1
C	4	6	1	2
D	10	20	1	2
E	1	2		2
F	2	2		1
G	6	16	1	2
H	6	9	1	1
I	2	5	1	3
J	1	1		1
K	2	2		1
L	14	30	1	2
M	11	32	1	2
N	3	5	1	2
O	1	1		1
P	1	1		1
Q	2	2		1
R	1	1		1
Total	74	147	1–7	1

**Table 2:** Fresh fishery product retailers managers’ socio-demographic data obtained from interviews.

Characteristics	*N* (%)
Sex	
Male	18 (24.3)
Female	56 (75.7)
Age	
30–39	7 (9.5)
40–49	17 (23)
50–59	21 (28.3)
60–69	18 (24.3)
70 or more	11 (14.9)
Nationality	
Portuguese	72 (97.3)
Other	2 (2.7)
Primary language	
Portuguese	74 (100)
Other	0
Education level	
Illiterate	5 (6.8)
1st cycle	22 (29.7)
2nd cycle	12 (16.2)
3rd cycle	21 (28.4)
High School	11 (14.9)
University level	3 (4)
Years working in fresh fishery products retail	
0–5	3 (4)
6–10	2 (2.7)
11–15	5 (6.8)
16–20	7 (9.4)
21–25	10 (13.5)
26–30	11 (14.9)
31–35	9 (12.2)
36–40	10 (13.5)
41–45	8 (10.8)
>46	9 (12.2)
Food handlers working in their fresh fishery products retail business	
1 to 2	57 (77)
3 to 5	12 (16.2)
>5	5 (6.8)
Contract with a food safety consultancy company	
Yes	21 (28.4)
No	53 (71.6)
Last training course on food hygiene, safety, and HACCP- based procedures	
Never had	5 (6.8)
Last 2 years	20 (27)
Between 2 to 5 years	27 (36.5)
Between 6 to 10 years	20 (27)
More than 10 years	2 (2.7)
Ongoing food safety management system supported by documents	
Yes	11 (14.9)
No	63 (85.1)

**Table 3:** Documental assessment of hand hygiene and food contact surfaces hygiene procedures compliance frequencies.

Requisites	Compliance	
	%	*n*
Personal hygiene program includes hand hygiene recommendations	6.8	5
Personal hygiene program mentions appropriate hand soap/sanitizer	2.7	2
Hand soap/sanitizer technical and safety data sheets included	5.4	4
Handwashing instructions displayed	9.5	7
Hygiene program includes food contact surfaces	8.1	6
Hygiene program considers:		
Specific food contact surfaces to be sanitized	8.1	6
Detergents/sanitizers to be used	2.7	2
Specific cleaning and sanitizing instructions	4.1	3
Detergent/sanitizer technical and safety data sheets included	2.7	2

**Table 4:** Hand hygiene practices compliance frequencies.

Requisites	Compliance	
	%	*n*
Hands and nails in adequate condition for food handling	58.1	43
Absence of hand and/or wrist adornments	28.4	21
Exclusive handwashing sink	25.7	19
Handwashing sink with touchless faucet	5.4	4
Handwashing performed with hot water	2.7	2
Hands are pre-rinsed with water	66.2	49
Hands are washed and rubbed with soap	1.4	1
Hand soap removal with running water	1.4	1
Appropriate handwashing duration	0	0
Hands dried with disposable paper towels	18.9	14
Use of sanitizer after handwashing	0	0
Disposable gloves used	8.1	6
Reusable gloves used	39.2	29
Reusable gloves sanitized after use	0	0
Correct handwashing before/after putting on/removing gloves	0	0

**Table 5:** Food contact surfaces hygiene practices compliance frequencies.

Requisites	Compliance	
	%	*n*
Suitable materials in food contact surfaces	100	74
Food contact surfaces maintained in good conditions	48.7	36
Pre-rinsing step applied	90.5	67
Recommended detergent application method	79.7	59
Sanitizing step applied	68.9	51
Recommended sanitizer application method	27	20
Arbitrary mix of detergent and sanitizer	47.3	35
Proper final rinsing	82.4	61
Adequate cleaning utensils	27	20
Chemicals stored in closed and identified place	29.7	22
Chemicals stored in original package	81.1	60
Chemicals properly labelled	70.3	52

## Data Availability

The datasets generated for this study are available on request to the corresponding author.

## Appendix A

**Table A1:** Checklist sections, sub-sections, and evaluated requisites.

**Sections**	**Questions**
Documental assessment of procedures	Hand hygiene: Does the personal hygiene program include hand hygiene recommendations? Is there a recommended handwashing soap/sanitizer in the personal hygiene program? Are there technical and safety data sheets for in-use handwashing soap and sanitizer? Are handwashing instructions available and easily displayed while performing the procedure? Food contact surfaces hygiene: Does the hygiene program consider food contact surfaces hygiene? The existing hygiene program for food contact surfaces considers the following: (i) surfaces to be sanitized; (ii) detergents/sanitizers to be used; (iii) detailed cleaning and sanitizing procedures. Are there technical and safety data sheets for the detergent and sanitizer used in food contact surfaces? Are surfaces cleaning and sanitizing work instructions available and easily displayed while performing the procedure?
Hand hygiene practices	Hand presentation: Do food handlers present their hands and nails in a suitable condition for food handling? Do food handlers present hand and/or wrist adornments? Handwashing equipment: Is there an exclusive handwashing sink for handwashing? Is there an automatic/non-hand operated water supply in the handwashing sink? Handwashing procedure: Is running water used to pre-rinse hands? Is hot water used for handwashing purposes? Is soap used in the handwashing procedure? Are hands and wrists fully washed with soap during the handwashing procedure? Is running water used to remove soap? Is a hand sanitizer used? Does the handwashing procedure take 40–60 s? Is hand drying appropriately performed? Are the in-use soap and sanitizer appropriate and specific for the handwashing procedure? Glove wearing: Are gloves used during working hours? Are disposable gloves changed whenever necessary? Are reusable gloves sanitized after use? Is the sanitizing procedure used for reusable gloves appropriate? Are hands washed correctly before/after putting on gloves?
Food contact surfaces hygiene practices	Surfaces materials and maintenance: Are food contact surfaces made of suitable materials? Are food contact surfaces in good conservation conditions? Surfaces hygiene procedure: Is a pre-rinsing step applied? Is a cleaning step applied? In the cleaning step, is the recommended detergent application method fully observed? Is a sanitizing step applied? In the sanitizing step, is the recommended sanitizer application method fully observed? Is the detergent mixed with the sanitizer prior to their application on surfaces? Are food contact surfaces appropriately rinsed in the end of the hygienization procedures? Cleaning utensils and chemicals management: Are the cleaning utensils adequate? Are all chemicals stored away from food handling areas in a closed and properly identified place? Are chemical products properly stored in their original package? Are chemical products properly labeled?

## References

[1] World Health Organization (WHO). https://www.who.int/health-topics/foodborne-diseases#tab=tab_1.

[2] European Food Safety Authority and European Centre for Disease Prevention and Control (2021). The European Union One Health 2019 Zoonoses Report. EFSA J..

[3] European Commission (2004). Commission Regulation (EC) No 852/2004 of 29 April 2004 on the hygiene of foodstuffs. Off. J. Eur. Union.

[4] European Commission (2004). Commission Regulation (EC) No 853/2004 of 29 April 2004 laying down specific hygiene rules for food of animal origin. Off. J. Eur. Union.

[5] European Commission (2016). Commission Notice 2016/C 278/01 of 30 July 2016 on the implementation of food safety management systems covering prerequisite programs and procedures based on the HACCP principles, including the facilitation/flexibility of the implementation in certain food businesses. Off. J. Eur. Union.

[6] Bucking M., Haugen J.E., Lelieveld H.L.M., Mostert M.A., Holah J. (2016). Improving hygiene control by sensors. Handbook of Hygiene Control in the Food Industry.

[7] van Asselt A.J., Giffel M.C., Lelieveld H.L.M., Mostert M.A., Holah J. (2016). Pathogen resistance to sanitisers. Handbook of Hygiene Control in the Food Industry.

[8] Luning P.A., Jacxsens L., Rovira J., Osés S.M., Uyttendaele M., Marcelis W.J. (2011). A concurrent diagnosis of microbiological food safety output and food safety management system performance: Cases from meat processing industries. Food Control.

[9] Lahou E., Jacxsens L., Daelman J., Van Landeghem F., Uyttendaele M. (2012). Microbiological Performance of a Food Safety Management System in a Food Service Operation. J. Food Prot..

[10] Notermans S., Powell S.C., Hoornstra E., Lelieveld H.L.M., Mostert M.A., Holah J. (2016). Introduction. Handbook of Hygiene Control in the Food Industry.

[11] Santos A., Cardoso M.F., Costa J., Gomes-Neves E. (2017). Meat Safety: An Evaluation of Portuguese Butcher Shops. J. Food Prot..

[12] Maillard J.-Y., Lelieveld H.L.M., Mostert M.A., Holah J. (2016). Testing the effectiveness of disinfectants and sanitisers. Handbook of Hygiene Control in the Food Industry.

[13] World Health Organization (WHO) WHO Guidelines on Hand Hygiene in Health Care—16 First Global Patient Safety Challenge—Clean Care Is Safer Care. https://www.who.int/gpsc/5may/tools/who_guidelines-handhygiene_summary.pdf.

[14] Michaels B. (2004). Understanding the Glove Risk Paradigm: Part I. Food Saf. Mag..

[15] Vitória A.G., Oliveira J.S.C., Almeida Pereira L.C., Faria C.P., São José J.F.B. (2021). Food safety knowledge, attitudes and practices of food handlers: A cross-sectional study in school kitchens in Espírito Santo, Brazil. BMC Public Health.

[16] Raaska L., Lelieveld H.L.M., Mostert M.A., Holah J. (2016). Managing contamination risks from food packaging materials. Handbook of Hygiene Control in the Food Industry.

[17] World Health Organization and Food and Agriculture Organization of the United Nations (2009). Codex Alimentarius: Food Hygiene (Basic Texts).

[18] Associação dos Comerciantes de Pescado (2012). Guia de Boas Práticas de Higiene para Produtos da Pesca Frescos, Grossistas e Retalhistas.

[19] European Commission (2003). Commission recommendation 2003/361/EC of 6 May 2003 concerning the definition of micro, small and medium-sized enterprises. Off. J. Eur. Union.

[20] Cunha D.T., Stedefeldt E., Rosso V.V. (2014). The role of theoretical food safety training on Brazilian food handlers’ knowledge, attitude and practice. Food Control.

[21] Tan S.L., Bakar F.A., Karim M.S.A., Lee H.Y., Mahyudin N.A. (2013). Hand hygiene knowledge, attitudes and practices among food handlers at primary schools in Hulu Langat district, Selangor (Malaysia). Food Control.

[22] Sibanyoni J.J., Tshabalala P.A., Tabit F.T. (2016). Food safety knowledge and awareness of food handlers in school feeding programmes in Mpumalanga, South Africa. Food Control.

[23] De Silva D.A.M. (2011). Faces of Women in Global Fishery Value Chains: Female Involvement, Impact and Importance in the Fisheries of Developed and Developing Countries.

[24] de Lisboa C.M. (2016). Plano Municipal dos Mercados de Lisboa 2016–2020.

[25] Barreta J. (2002). Organização e Gestão dos Mercados Municipais. Mudar e Inovar para Competir.

[26] Soares L.S., Almeida C., Cerqueira E., Carvalho J., Nunes I. (2012). Knowledge, attitudes and practices in food safety and the presence of coagulase-positive staphylococci on hands of food handlers in the schools of Camaçari, Brazil. Food Control.

[27] Liu S., Liu Z., Zhang H., Lu L., Liang J., Huang Q. (2015). Knowledge, attitude and practices of food safety amongst food handlers in the coastal resort of Guangdong, China. Food Control.

[28] Lee H., Abdul Halim H., Thong K., Chai L. (2017). Assessment of Food Safety Knowledge, Attitude, Self-Reported Practices, and Microbiological Hand Hygiene of Food Handlers. Int. J. Environ. Res. Public Health.

[29] Agüeria D.A., Terni C., Baldovino V.M., Civit D. (2018). Food safety knowledge, practices and attitudes of fishery workers in Mar del Plata, Argentina. Food Control.

[30] Praia E.F., Henriques A.R. (2021). Assessing the implementation of food defense requirements in industrial meat-based food processors. Braz. J. Food Technol..

[31] Visser T., van Scheers L. (2018). Can family business managers manage family business risks?. J. Contemp. Manag. Issues.

[32] Osaili T.M., Jamous D.O., Obeidat B.A., Bawadi H.A., Tayyem R.F., Subih H.S. (2013). Food safety knowledge among food workers in restaurants in Jordan. Food Control.

[33] Akabanda F., Hlortsi E.H., Owusu-kwarteng J. (2017). Food safety knowledge, attitudes and practices of institutional food-handlers in Ghana. BMC Public Health.

[34] De Oliveira C.A., da Cruz A.G., Tavolaro P., Corassin C.H., Barros-Velazquez J. (2016). Food Safety: Good manufacturing practices (GMP), sanitation standard operating procedures (SSOP), hazard analysis and critical control point (HACCP). Antimicrobial Food Packaging.

[35] Askarian M., Kabir G., Aminbaig M., Memish Z., Jafari P. (2004). Knowledge, Attitudes, and Practices of Food Service Staff Regarding Food Hygiene in Shiraz, Iran. Infect. Control Hosp. Epidemiol..

[36] Aarnisalo K., Tallavaara K., Wirtanen G., Maijala R., Raaska L. (2006). The hygienic working practices of maintenance personnel and equipment hygiene in the Finnish food industry. Food Control.

[37] Powell D.A., Jacob C.J., Chapman B.J. (2011). Enhancing food safety culture to reduce rates of foodborne illness. Food Control.

[38] European Parliament (2006). Regulation (EC) No 1907/2006 of 18 December 2006 concerning the Registration, Evaluation, Authorisation and Restriction of Chemicals (REACH), establishing a European Chemicals Agency, amending Directive 1999/45/EC and repealing Council Regulation (EEC) No 793/93 and Commission Regulation (EC) No 1488/94 as well as Council Directive 76/769/EEC and Commission Directives 91/155/EEC, 93/67/EEC, 93/105/EC and 2000/21/EC. Off. J. Eur. Union.

[39] Ramos-Peralonso M.J., Wexler P. (2014). Chemical Hazard Communication and Safety Data Sheets. Encyclopedia of Toxicology.

[40] Dzwolak W. (2019). Assessment of HACCP plans in standardized food safety management systems—The case of small-sized Polish food businesses. Food Control.

[41] Al Suwaidi A.H.E., Hussein H., Al Faisal W., El Sawaf E., Wasfy A. (2015). Hygienic Practices Among Food Handlers in Dubai. Int. J. Prev. Med. Res..

[42] Garayoa R., Vitas A.I., Díez-Leturia M., García-Jalón I. (2019). Food safety and the contract catering companies: Food handlers, facilities and HACCP evaluation. Food Control.

[43] Mattick K., Durham K., Hendrix M., Slader J., Griffith C., Sen M., Humphrey T. (2003). The microbiological quality of washing-up water and the environment in domestic and commercial kitchens. J. Appl. Microbiol..

[44] Todd E., Michaels B.S., Smith D., Greig J.D., Bartleson C.A. (2010). Outbreaks Where Food Workers Have Been Implicated in the Spread of Foodborne Disease. Part 9. Washing and Drying of Hands To Reduce Microbial Contamination. J Food Prot..

[45] United States of America Centers for Disease Control and Prevention. https://www.cdc.gov/fdoss/pdf/2016_FoodBorneOutbreaks_508.pdf.

[46] Hedberg C.W. (2013). Explaining the Risk of Foodborne Illness Associated with Restaurants: The Environmental Health Specialists Network (EHS-Net). J. Food Prot..

[47] Sprenger R.A. (2017). Hygiene for Management a Text for Food Hygiene Courses.

[48] Baş M., Ersun A.S., Kıvanç G. (2006). The evaluation of food hygiene knowledge, attitudes, and practices of food handlers’ in food businesses in Turkey. Food Control.

[49] Valero A., Rodríguez M.-Y., Posada-Izquierdo G.D., Pérez-Rodríguez F., Carrasco E., García-Gimeno R.M., Makun H.A. (2016). Risk Factors Influencing Microbial Contamination in Food Service Centers. Significance, Prevention and Control of Food Related Diseases.

[50] Green L.R., Radke V., Manson R., Bushnell L., Reimann D.W., Mack J.C., Motsinger M.D., Stigger T., Selman C.A. (2007). Factors Related to Food Worker Hand Hygiene Practices. J. Food Prot..

[51] Freitas J.F., Calazans D., Alchieri J. (2014). Food handlers’ occupational and professional training characterization. J. Nutr. Food Sci..

[52] Brown L.G., Le B., Wong M.R., Reimann D., Nicholas D., Faw B., Davis E., Selman A.C. (2014). Restaurant manager and worker food safety certification and knowledge. Foodborne Pathog. Dis..

[53] Zanin L.M., Da Cunha D.T., De Rosso V.V., Dias Capriles V., Stedefeldt E. (2017). Knowledge, attitudes and practices of food handlers in food safety: An integrative review. Food Res. Int..

[54] Jullien C., Bénézech T., Carpentier B., Lebret V., Faille C. (2003). Identification of surface characteristics relevant to the hygienic status of stainless steel for the food industry. J. Food Eng..

[55] Gkana E., Lianou A., Nychas E. (2016). Transfer of Salmonella enterica Serovar Typhimurium from Beef to Tomato through Kitchen Equipment and the Efficacy of Intermediate Decontamination Procedures. J. Food Prot..

[56] Holah J.T., Thorpe R.H. (1990). Cleanability in relation to bacterial retention on unused and abraded domestic sink materials. J. Appl. Bacteriol..

[57] Boulangé-Petermann L. (1996). Processes of bioadhesion on stainless steel surfaces and cleanability: A review with special reference to the food industry. Biofouling.

[58] Matthewson L., Heacock H. (2017). Methods for cleaning & sanitizing food contact surfaces (countertops) to prevent cross contamination in restaurant kitchens. BCIT Environ. Public Health J..

[59] Hernández-Navarrete M.J., Celorrio-Pascual J.M., Lapresta Moros C., Solano Bernad V.M. (2014). Fundamentos de antisepsia, desinfección y esterilización. Enferm. Infecc. Microbiol. Clínica.

[60] Andrade J.C., João A.L., Alonso C.d.S., Barreto A.S., Henriques A.R. (2020). Genetic Subtyping, Biofilm-Forming Ability and Biocide Susceptibility of Listeria monocytogenes Strains Isolated from a Ready-to-Eat Food Industry. Antibiotics.

[61] Henriques A.R., Fraqueza M.J., Viccario T. (2015). Listeria monocytogenes and ready-to-eat meat-based food products: Incidence and control. Listeria Monocytogenes: Incidence, Growth Behavior and Control.

